# Old drug, new trick: proton pump inhibitors find new purpose in cancer care

**DOI:** 10.18632/oncotarget.28053

**Published:** 2021-09-14

**Authors:** Natalie A. Ridge, Mohit Shiv Agarwal, Mohamad Hassan Fakhreddine

**Keywords:** radiosensitizer, proton pump inhibitor, esomeprazole, radioresistance, radiotherapy

***News on:** Esomeprazole enhances the effect of ionizing radiation to improve tumor control by Hebert et al. Oncotarget. 2021; 12:1339–53. https://doi.org/10.18632/oncotarget.28008. [PubMed]*

More than half of all cancer patients undergo radiation as part of their treatment. Unfortunately, multiple factors contribute to radiation resistance within solid tumors, and can lead to treatment failure. Radiosensitizing chemotherapies such as cisplatin are often used in conjunction with radiotherapy to enhance tumor cell killing. However, such agents are non-selective and as such can cause a wide array of significant side effects. Therefore, there is a great need to identify novel strategies to improve radiation therapeutic ratios and enhance efficacy, while limiting non-selective toxicity.

We read with great interest the study by Hebert et al. recently published in Oncotarget, which investigates the radiosensitizing effect of the widely used over-the-counter proton pump inhibitor (PPI) esomeprazole. Major obstacles to *de novo* development of new cancer drugs include high cost and risk of failure, which can slow the process. Repurposing of existing drugs, such as esomeprazole, for new therapeutic indications is a resourceful alternative strategy to accelerate and expand access for patients to new treatment options. PPIs, which are widely available and have a well-characterized safety profile, have emerged as a promising adjunct to standard cancer treatments. Recently published [1, 2] and ongoing clinical studies have suggested that PPIs can improve cancer outcomes in patients. Prior preclinical studies have demonstrated their ability to selectively inhibit cancer cell proliferation and induce cytotoxicity, as well as enhance the efficacy of various chemotherapeutic agents [3–5]. Additionally, and very interestingly, esomeprazole has previously been found to mitigate normal tissue toxicity from chemotherapy and radiation [6, 7].

In the present study, Hebert et al. not only demonstrate the anti-cancer activity of esomeprazole but offer new compelling evidence for the radiosensitizing effect of PPIs. The authors report *in vitro* and *in vivo* results showing that esomeprazole enhances cell killing, antiproliferative, and tumor control effects of radiation, without causing non-specific cytotoxicity. They further reveal through mechanistic studies that esomeprazole appears to have pleiotropic anti-cancer activity. These include but are not limited to: arresting cancer cells at the G0/G1 phase of the cell cycle through upregulation of p21 to block proliferation, and amplifying radiation-induced DNA damage by impairing DNA repair.

In the context of solid tumors, altered energy metabolism, hypoxia, increased expression and activity of proton pumps, and infiltrating inflammatory immune cells contribute to creating an acidic extracellular microenvironment, and relative intracellular alkalinization. This pH homeostasis promotes tumor proliferation, invasion, immune escape, and resistance to chemotherapy/radiotherapy. It has been discussed previously that drugs targeting cellular proton pump systems, including PPIs, may act in-part to attenuate resistance to standard treatment modalities by reversing this pH gradient [8, 9]. Interestingly, in the present study, *in vitro* experiments with alternative antacids of the H2-receptor antagonist class did not demonstrate growth inhibition, suggesting that the anti-cancer activity observed here was likely independent from regulation of intracellular pH or buffering of the culture media. Further, culture assays were conducted under physiologic pH, indicating that at least in this context, prodrug activation under acidic conditions does not underlie PPI anti-cancer activity. However, pH-dependent drug activation/modification may at least in part explain the differential effects in cancer versus normal cells.

As PPIs are clearly capable of influencing multiple molecular events and cellular activities, it is likely that the physiological responses underlying the benefits observed in cancer patients are multifactorial. Further exploration of the anticancer and tissue protective mechanisms of PPIs in cancer patients may reveal key modifiers of clinical outcomes. Hebert et al’s preclinical study findings serve as a solid base for the design of clinical trials utilizing the PPI esomeprazole in conjunction with radiotherapy. If such trials are successful, the potential for a widely available, repurposed drug with the dual function of tumor radiosensitization and normal tissue protection would be a meaningful advance in oncology. We look forward to reading about the outcomes of such trials in the future.
